# Novel Use of Matched Filtering for Synaptic Event Detection and Extraction

**DOI:** 10.1371/journal.pone.0015517

**Published:** 2010-11-24

**Authors:** Yulin Shi, Zoran Nenadic, Xiangmin Xu

**Affiliations:** 1 Department of Anatomy and Neurobiology, School of Medicine, University of California Irvine, Irvine, California, United States of America; 2 Department of Biomedical Engineering, University of California Irvine, Irvine, California, United States of America; University of California, United States of America

## Abstract

Efficient and dependable methods for detection and measurement of synaptic events are important for studies of synaptic physiology and neuronal circuit connectivity. As the published methods with detection algorithms based upon amplitude thresholding and fixed or scaled template comparisons are of limited utility for detection of signals with variable amplitudes and superimposed events that have complex waveforms, previous techniques are not applicable for detection of evoked synaptic events in photostimulation and other similar experimental situations. Here we report on a novel technique that combines the design of a bank of approximate matched filters with the detection and estimation theory to automatically detect and extract photostimluation-evoked excitatory postsynaptic currents (EPSCs) from individually recorded neurons in cortical circuit mapping experiments. The sensitivity and specificity of the method were evaluated on both simulated and experimental data, with its performance comparable to that of visual event detection performed by human operators. This new technique was applied to quantify and compare the EPSCs obtained from excitatory pyramidal cells and fast-spiking interneurons. In addition, our technique has been further applied to the detection and analysis of inhibitory postsynaptic current (IPSC) responses. Given the general purpose of our matched filtering and signal recognition algorithms, we expect that our technique can be appropriately modified and applied to detect and extract other types of electrophysiological and optical imaging signals.

## Introduction

Neurons in the brain and nervous system in general communicate with one another by forming connections mostly through synapses. Typical neurophysiological studies involve experimental recordings from many neurons, and may require detailed examination and analysis of synaptic events. For example, laser scanning photostimulation experiments are effective for mapping local circuit inputs to individually recorded neurons [1], [2], [3], as simultaneous whole-cell recordings from a postsynaptic neuron with photostimulation of clusters of presynaptic neurons (via glutamate uncaging) at many different locations provide quantitative measures of spatial distribution of excitatory and inhibitory inputs impinging onto individually recorded neurons. Similar to most other synaptic physiological analyses, photostimulation data analysis involves identification and detection of hundreds of response traces that are recorded from each individual cell. Although photostimulation maps of synaptic inputs can be constructed by simply averaging postsynaptic current amplitudes within a response window [2], [3], [4], [5], a more comprehensive understanding of synaptic connectivity requires detection of individual synaptic events and measurement of parameters such as event occurrence times, amplitudes and frequencies [6], [7]. While the human detection of these events is typically aided by software applications, the process is still laborious and time-consuming, which precludes efficient treatment of large datasets [7], [8].

As automated detection of synaptic events is of practical importance to experimental neuroscience, several different approaches (particularly for detection of spontaneous synaptic events) have been developed, where detection algorithms are based upon amplitude thresholding, and fixed or scaled template matching [9], [10], [11], [12], [13], [14]. An unpublished method (http://huguenard-lab.stanford.edu/public/) noted in [5] could detect photostimulation-evoked EPSCs based upon the estimated EPSC differentiation window sizes and event amplitudes, which need to be carefully adjusted and tested on recorded traces of each map to ensure detection of synaptic events. However, these algorithms are found to be of limited utility for detection of signals with variable amplitudes and superimposed events that have complex waveforms [10], [12]; thus they are not optimal for detection of evoked synaptic events in photostimulation and other similar experimental situations.

In the present study we introduce a novel technique for detection and extraction of photostimulation-evoked excitatory postsynaptic currents (EPSCs) from individually recorded neurons in cortical circuit mapping experiments. Our technique is motivated by the observation that a matched filter represents a detector that maximizes the signal-to-noise ratio (SNR) [15]. In other words, if a noisy time series is match-filtered, the time samples that contain a signal of interest are amplified while those containing noise are suppressed, which then facilitates the separation of signal and noise in the filtered time series. To synthesize such a filter, the signal to be detected must be perfectly known so that the filter can be matched to the signal, which is not possible in most experimental situations. To circumvent this constraint, our technique starts with a training stage, where several high-SNR EPSCs are identified by a human operator and fitted by polynomial models to build an array (bank) of approximate matched filters (templates). The filter bank provides a rich class of waveforms that potentially match those of EPSCs found in experimental recordings, thereby increasing the likelihood of their detection. In the fully automated detection stage, experimental data traces are filtered in the time domain with the polynomial templates obtained in the training stage. This amounts to convolving the data traces to be analyzed with the templates, with candidate EPSCs having a better match with the templates and thus yielding larger convolution amplitudes. To detect EPSCs, the convolution traces are then compared to an event detection threshold and candidate EPSCs are localized and extracted by using statistical parameters estimated in the training stage.

The paper presents novel EPSC detection and extraction algorithms, as well as technical implementation details. The sensitivity and specificity of the method were first evaluated on simulated data, and subsequently validated on experimental data by comparing its performance to that of visual event detection performed by human operators. We also extended this method to the detection and analysis of inhibitory postsynaptic current (IPSC) responses. Finally, this new technique was applied to quantify and compare photostimulation-evoked EPSCs obtained from excitatory pyramidal cells and fast-spiking interneurons.

## Materials and Methods

### Experimental recordings

Wild-type C57/B6 mice were used in the experiments. All animals were handled and experiments were conducted in accordance with the protocol (#2008-2796) approved by the Institutional Animal Care and Use Committee at the University of California, Irvine. To prepare living brain slices, animals (postnatal day 17–23) were deeply anesthetized with pentobarbital sodium (>100 mg/kg, i.p.), rapidly decapitated, and their brains were removed. Coronal sections of prefrontal cortex were cut 400 µm thick with a vibratome (VT1200S, Leica Systems) in sucrose-containing artificial cerebrospinal fluid (ACSF) (in mM: 85 NaCl, 75 sucrose, 2.5 KCl, 25 glucose, 1.25 NaH2PO4, 4 MgCl2, 0.5 CaCl2, and 24 NaHCO3). Slices were first incubated in sucrose-containing ACSF for 30 min to 1 h at 32°C, and then transferred to recording ACSF (in mM: 126 NaCl, 2.5 KCl, 26 NaHCO3, 2 CaCl2, 2 MgCl2, 1.25 NaH2PO4, and 10 glucose) at room temperature. Throughout incubation and recording, the slices were continuously bubbled with 95% O2-5% CO2.

Cortical slices were visualized with an upright microscope (BW51X, Olympus) with infrared differential interference contrast optics. Electrophysiological recordings, photostimulation, and imaging of the slice preparations were done in a slice perfusion chamber mounted on a motorized stage of the microscope. An aliquot of MNI-caged-L-glutamate (4-methoxy-7-nitroindolinyl-caged L-glutamate, Tocris Bioscience, Ellisville, MO) was added to 20–25 ml of circulating ACSF for a concentration of 0.2 mM caged glutamate. To perform whole cell recording, cells were visualized at high magnification (60× objective, 0.9 NA; LUMPlanFl/IR, Olympus). Neurons were patched with borosilicate electrodes and recorded at room temperature. The patch pipettes (4–6 MΩ resistance) were filled with an internal solution containing (in mM) 126 K-gluconate, 4 KCl, 10 HEPES, 4 ATP-Mg, 0.3 GTP-Na, and 10 phosphocreatine (pH 7.2, 300 mOsm). For some recordings in which IPSCs were measured, potassium in the internal solution was replaced with cesium. The internal solution also contained 0.1% biocytin for cell labeling and morphological identification. Once stable whole cell recordings were achieved with good access resistance (usually <20 MΩ), the microscope objective was switched from 60× to 4× for laser scanning photostimulation. At low magnification (4× objective lens, 0.16 NA; UplanApo, Olympus), the slice images were acquired by a high-resolution digital CCD camera (Retiga 2000, Q-imaging, Austin, TX) and used for guiding and registering photostimulation sites in cortical slices.

The design of our laser scanning photostimulation system has been described previously [16]. A laser unit (model 3501, DPSS Lasers, Santa Clara, CA) was used to generate a 355 nm UV laser for glutamate uncaging. Various laser stimulation positions were achieved through galvanometer-driven X-Y scanning mirrors (Cambridge Technology, Cambridge, MA), as the mirrors and the back aperture of the objective were in conjugate planes, thereby translating mirror positions into different scanning locations at the objective lens focal plane. During mapping experiments, photostimulation was applied to 16×16 patterned sites (centered at the recorded neuron) in a nonraster, nonrandom sequence, while whole-cell voltage-clamp recordings were made from the recorded postsynaptic neurons with EPSCs and IPSCs measured at the holding potential of −70 mV and 0 mV, respectively, across photostimulation sites. Data were acquired with a Multiclamp 700B amplifier (Molecular Devices, Sunnyvale, CA), data acquisition boards (models PCI MIO 16E-4 and 6713, National Instruments, Austin, TX), and custom-modified version of Ephus software (Ephus, available at https://www.ephus.org/). Data were low-pass filtered at 2 kHz using a Bessel filter, digitized at 10 kHz, and stored on a computer. For more detailed electrophysiology and photostimulation procedures, please refer to previously published studies [2], [16].

### Design of matched filters, and EPSC detection and extraction algorithms

Our detection method consists of two stages: (i) the design of matched filters (templates); and (ii) the fully automated event detection with established filters (see Figure S1 for an explanatory flow chart). In the filter design stage, referred to as the training stage, the user presents the algorithm with examples of identified EPSCs, based on which templates and statistical parameters of their waveforms are estimated and stored. In the detection stage, the templates and parameters obtained in the training stage are used to detect EPSCs.

#### Training Stage

In the training stage, the user identifies raw experimental data traces that contain evoked EPSCs that are sufficiently strong with respect to background noise; and the user is prompted to manually mark evoked EPSCs. This procedure typically involves sequential selection of several synaptic responses with different shapes, durations and amplitudes. The onset of the EPSC should be taken as the point where the signal starts falling sharply from the baseline. Similarly, the offset point should be the point where the signal returns to baseline. The onset and offset points should be at a similar baseline level. If this condition is violated (presumably due to a high noise level or direct response contamination), it is recommended that a new EPSC be used for training. Superimposed EPSCs are not appropriate to be used for training. An 8-th order polynomial model is fitted through the segment of each identified EPSC, normalized by its L_1_ norm [The L1 norm of a vector 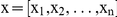 is defined as: 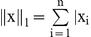], and saved as a template (approximate matched filter) for the detection stage. The normalization is necessary to minimize the dependence of EPSC detectability on the filter amplitude. While EPSCs from a single synaptic event are typically modeled using exponential functions [14], [17], [18], [19], most of photostimulation-evoked EPSCs appear to represent compound responses of multiple synapses, and the exponential models proved inadequate. The 8-th order polynomial model, however, provided an excellent fit, given the sampling rate of 10 kHz and the mean duration of EPSCs of ∼14 ms. While our method and its software implementation allow the user to change the order of the model, choosing polynomials of higher order may result in overfitting. Several parameters are then calculated and stored for further analysis, including the duration of the EPSCs, defined by manual mouse clicks, and the duration of the leading and trailing parts of EPSCs, defined as the absolute value of the difference between the time of the (negative) peak of the selected EPSC and its onset and offset, respectively. In addition, the amplitude of the selected EPSC, defined as the difference of the amplitude value at the onset and peak time of the EPSC, is calculated. Finally, the selected raw EPSC is convolved with the yielded template and the maximum value of the convolution signal, c_max_, is logged. The purpose of this step is to obtain the statistics of convolution amplitudes for the detection stage. Due to the presence of noise and the fact that EPSCs are asymmetric, there is typically a time shift between the peaks of convolution and EPSC traces, and the shift value is also recorded and stored. The role of these parameters will be precisely defined in the detection stage. The whole procedure is then repeated with a different trace or a different EPSC within the same trace, which amounts to building a bank of approximate matched filters for the detection stage. A minimum recommended number of filters in the bank is 10, although 18 filters were used in the present study. In addition to increasing the likelihood of detecting EPSCs with various shapes and durations, multiple templates allow the statistics of the above parameters to be estimated more accurately. Subsequently, based on these parameters, detection thresholds and safeguards against false detection can be set in a statistically meaningful manner. It should be noted that the user's involvement only includes selecting EPSCs with mouse clicks, and that all subsequent calculations are automated. Typical time necessary to obtain the filter bank and the associated parameter statistics is less than 10 min. It should also be noted that templates and parameters trained on a data set from one experiment can often be used for detection of EPSCs in other similar experiments.

#### Detection Stage

In the detection stage, the arrival (occurrence) times of candidate EPSCs are found and processed, and short segments of data around the estimated occurrence times are extracted for further analysis. Specifically, the data trace under investigation is first high-pass filtered (>10 Hz) with a 5^th^ order, infinite impulse response Butterworth filter (see Figure S2). The role of this filter is to minimize the effect of the direct uncaging response (see Results), whose duration is much longer than that of synaptically mediated indirect responses (EPSCs). To minimize the phase distortions, this filter is implemented as a zero-phase forward and reverse digital filter [20]. The high-pass filtered signal is then convolved with all the filters from the bank, and the convolution traces (one for each filter) are time-shifted to minimize the difference between the time of the convolution peak and a potential EPSC peak, and thus facilitate a more precise estimation of EPSCs' occurrence times. The applied time shifts are those recorded in the training stage (see above). Time-shifted convolution traces are then compared to a detection threshold. For experimental data, this threshold is typically chosen between −1.5*σ* and *μ*−*σ*, where *μ* is the mean value of c_max_ obtained in the training stage, and *σ* is its standard deviation. The program allows the user to change the detection threshold, should it be necessary. The points of threshold crossing represent potential arrival times of EPSCs with two exceptions. First, *W_d_* ms within the onset of the laser stimulus, no synaptic responses are expected to be found (see below), and our method dismisses any potential events within this window. The default value for *W_d_* is 10 ms. Second, *W_o_* ms (*W_o_* = 30 ms by default) within the laser stimulus, convolution traces may still be affected by the direct response, yielding extremely large values. Therefore, the convolution traces within this window are compared to an additional (outlier) threshold, e.g. chosen as *μ*+4*σ*, where *μ* and *σ* are defined as above, and potential EPSCs whose convolution traces exceed this threshold are dismissed (see Figure S2). For each convolution trace, the samples that exceed the detection threshold form the so-called suprathreshold time segments. Within each eligible suprathreshold segment outside of *W_d_* time window, the center of mass of each convolution trace is found and declared as an occurrence time candidate, *t_cm_*, of an EPSC.

To localize EPSCs, the occurrence time candidates are processed from earlier to later along the original data trace in the following manner. First, for each potential EPSC, its negative peak is found in the vicinity of the occurrence time candidate, *t_cm_*, defined as 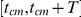, where *L* (e.g., 4.4 ms) and *T* (10.2 ms) are the mean leading and trailing parts of EPSCs estimated from the training stage. If multiple negative peaks are found around an occurrence time, they are scored according to several criteria, and the peak with the highest score is selected (see Figure S3). The location of the peak, *t_p_*, is then taken as the estimated EPSC occurrence time. Its onset and offset times are further identified within the segment 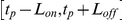. Here 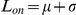 where *μ* and *σ* are the mean and standard deviation of the leading part of EPSC estimated in the training stage, and 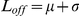 where *μ* and *σ* are the mean and standard deviation of the trailing part of EPSC estimated in the training stage. Specifically, as illustrated in Figure S4, the onset point of this potential EPSC is found as the largest positive peak on the segment 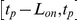; and the offset point is located as the largest local peak within the trailing part of the EPSC waveform between *t_p_* and *t_p_+L_off_*, or between *t_p_* and the onset of the next potential EPSC within 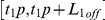. An additional measure is employed to detect potential overlapping EPSC events within 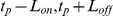 (see Figure S4). The potential EPSCs are required to exceed an amplitude threshold based upon the mean amplitude of spontaneous EPSCs assessed in the training stage. All the above procedures are repeated to process all occurrence time candidates to detect and localize potential EPSCs. Short segments of detected events centered at 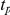 are then extracted and saved for further analysis. EPSC parameters, such as the peak amplitude (defined as the difference between the amplitudes at t_p_ and the onset), the summed input (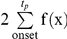), and the number of detected events, are subsequently calculated and analyzed.

### Modeling Neural Data

Simulated neural data were used to evaluate the performance of our method. To mimic experimental conditions, 10 EPSCs from actual whole-cell recording experiments were detected by a human operator, normalized to the amplitude of the largest EPSC (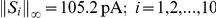) [The L_∞_ norm of a vector of a vector 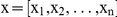 is defined as: 
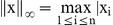
] and stored in a test template library. The rationale for this normalization will be explained below. For each trial, a Poisson process with the mean rate of 20 events per second and a refractory period of 27 ms was used to generate a sequence of EPSC arrival times. To account for overlapping events, in 20% of cases the refractory period was ignored. With a maximum duration of these test templates being ∼20 ms, this Poisson event generator produced overlapping events with reasonable intervals, as seen in real experimental recordings. The duration of each trial was set to 215 ms, with an average of 4.28 EPSCs to be generated. For each trial, the test templates were drawn at random from the library (with a uniform distribution) and centered at the arrival times generated by the Poisson process above to form a train of test templates.

To model the noise, some 160 whole-cell recordings that did not yield any evoked response (as established by the visual inspection by a human operator) were identified, normalized (mean: 0, standard deviation: 1) and saved in a noise template library. The duration of these traces was 400 ms. Note that these traces contain spontaneous activity, which presents realistic challenges to our detection method by creating potential false alarms. Other advantages of this noise model over traditionally used autoregressive models that rely on spectrum fitting are discussed at length in previous studies [21], [22], [23]. For each trial with a given SNR, defined here as 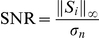, where *σ_n_* is the desired noise standard deviation, a 215-ms-long noise segment was selected randomly from the noise library, scaled to the desired SNR (i.e. multiplied by 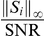) and added to the train of test templates. The normalization of test templates admits description of each trial with a single SNR, for otherwise SNRs need to be averaged over multiple events. Note that the average SNR is not a perfect measure of noisiness of the data as two trials with the same SNR may pose vastly different challenges to the detection algorithm [21]. Note that despite the normalization of the test templates, the detection of EPSCs with variable amplitudes can be effectively simulated by varying SNRs.

For analysis of the model data, 200 Monte Carlo trials were generated for each SNR, the threshold was varied, and the detection technique with established matched filters was applied. The results are shown as receiver operating characteristic (ROC) curves, illustrating the probability of correct detection (Pcd) and the probability of false alarm (Pfa). The detection of EPSC test template was declared correct if the absolute value of the difference between estimated and true arrival times was ≤1.5 ms. Note that this tolerance is significantly smaller than the average duration of test template EPSCs (∼11 ms). If no EPSC was detected within 1.5 ms of the true arrival time, an omission was declared. Similarly, if no true arrival time is found within 1.5 ms of the estimated arrival time, a false alarm was declared. To calculate Pcd and Pfa, instances of correct detections and false alarms are counted on a trial-by-trial basis, and averaged over trials. Please see [21] for the details of our averaging methodology.

### Software programming

All programming and data processing was done in MATLAB 2008 running on a Windows 7 PC laptop computer, with a 2.4 GHz Core 2 Duo processor and 4 GB of RAM. Once the matched filters are established, the automated detection and measurements of EPSCs in one typical data set containing 256 data traces (1 second length, sampled at 10 kHz) only requires a minute or so. A basic tutorial and software implementation of our technique will be publicly available at the authors' webpage.

## Results

### Detection of photostimulation-evoked synaptic events through matched filtering

**Figure 1:**
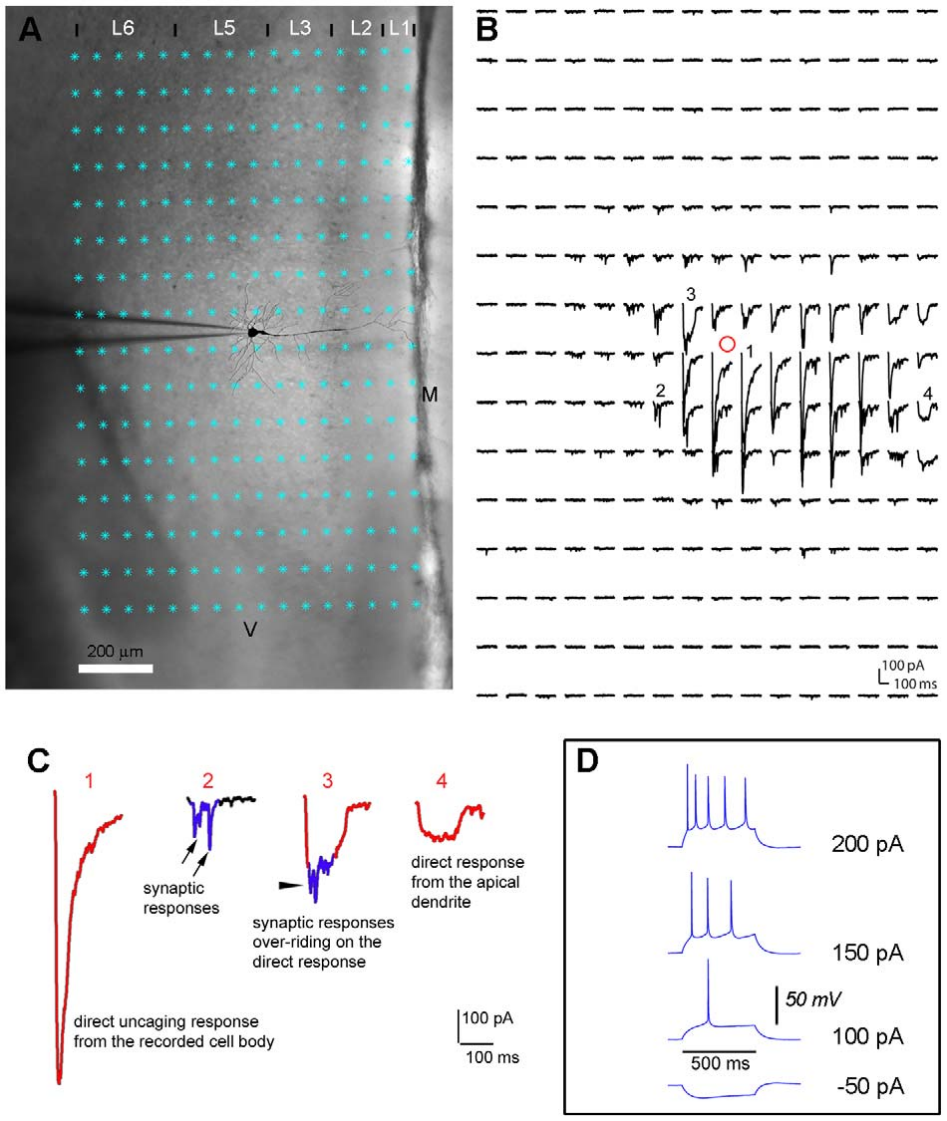
Laser scanning photostimulation combined with whole cell recordings to map local circuit input to an excitatory pyramidal neuron. A shows a mouse prefrontal cortical slice image with the superimposed photostimulation sites (16×16 cyan stars, spaced at 60 µm×100 µm) across all the cortical layers 1, 2, 3, 5 and 6 (i.e., L1–L6). Note that the prefrontal cortex lacks granular layer 4 found in primary sensory cortex. The glass electrode was recording from an excitatory pyramidal neuron (shown with a scaled reconstruction with major dendrites) in upper layer 5 of the prelimbic area in prefrontal cortex. M denotes medial, and V denotes ventral. B shows an array of photostimulation-evoked response traces from most locations shown in A, with the cell held at −70 mV in voltage clamp mode to detect inward excitatory synaptic currents (EPSCs). The red circle indicates the cell body location. Only the 200 ms of the recorded traces after the onset of laser photostimulation (1 ms, 25 mW) are shown. Different forms of photostimulation responses are illustrated by the traces of 1, 2, 3 and 4, which are expanded and separately shown in C. Trace 1 is an example of the direct response (shown in red) to glutamate uncaging on the cell body. Trace 2 is a typical example of synaptic input responses (blue). Trace 3 shows synaptic responses (blue) over-riding on the relatively small direct response (red) evoked from the cell's proximal dendrites. Trace 4 is another form of direct response (red) evoked from apical dendrites. D shows the pyramidal cell's intrinsic firing pattern with its voltage response traces to current injections at amplitudes of -50, 100, 150 and 200 pA, respectively.

Overall, photostimulation-evoked EPSCs represent a range of complex synaptic events that may be encountered in other studies of synaptic connections using focal electrical stimulation and dual or multiple intracellular recordings in highly localized circuits formed by neurons of high connection probabilities [13], [24], [25]. As illustrated in Figure 1, photostimulation can induce two major forms of excitatory responses: (1) direct glutamate uncaging responses (direct activation of the recorded neuron's glutamate receptors); and (2) synaptically mediated responses (EPSCs) resulting from the suprathreshold activation of presynaptic excitatory neurons. Responses within the 10 ms window from laser onset were considered direct, as they had a distinct shape (longer rise time) and occurred immediately after glutamate uncaging (shorter latency) (Figure 1C). Synaptic currents with such short latencies are not possible because they would have to occur before the generation of action potentials in photostimulated neurons [2], [7], [8], [16]. Therefore, direct responses need to be excluded from local synaptic input analysis. However, at some locations, synaptic responses were over-riding on the relatively small direct responses and they needed to be identified and included in synaptic input analysis (Figure 1C). Detection and extraction of this type of synaptic events actually presents a major challenge for automatic signal detection and extraction using algorithms in previously published techniques. In addition, synaptically-mediated responses have varying amplitudes and frequencies with overlapping EPSC events.

**Figure 2:**
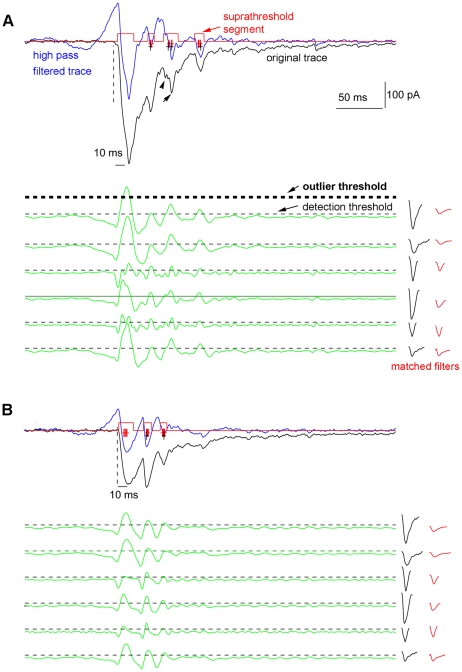
Detection of EPSCs with matched filters. A. In the top portion, the original and high-pass filtered traces are aligned. The raw trace, which is high-pass filtered with a Butterworth filter, contains a large direct response and synaptically mediated responses. The vertical dashed line indicates the photostimulation laser onset. The direct response window is defined as 10 ms within the laser onset. The filtered data trace is convolved with all the filters (a total of 18 matched filters in this case) from the bank, and the convolution traces (one for each filter) are compared to a threshold. In the bottom portion of A are shown 6 example convolution traces (green) produced with 6 matched filters (red) and their original EPSC templates (black). The detection threshold (dashed) is chosen as μ−1.2σ (11 pA), where *μ* is the mean value (28.4 pA) of *c*_max_ obtained in the training stage from the bank of 18 filters and *σ* is its standard deviation (14.5 pA). All the samples of the convolution traces that cross the detection threshold form the supratheshold segments (red squares); each trace may has its own set of suprathreshold segments. The red crosses illustrate the centers of mass of the supratheshold segments and represent potential EPSC occurrence times, while the black crosses are determined as identified EPSC peaks. As the arrow heads point out, more than one EPSCs can be identified within one suprathreshold segment. As the convolution values of the direct response are large and exceed the outlier threshold, defined as μ+4 σ (86 pA) within *W* ms (i.e., 30 ms) after the laser onset, the direct response is not detected as an EPSC response. B is similarly formatted as A, and shows another example to detect both a direct response and synaptically mediated responses. The direct response in B is relatively small, and its peak values of the convolution traces do not exceed the outlier threshold. But the response is correctly identified as a direct response, because the leading edge of the response is located within the 10 ms direct response window.

Our new technique of matched filtering can be effectively applied to detection of photostimulation-evoked EPSCs, as exemplified in Figure 2. The raw data trace was first high-pass filtered with a Butterworth filter, which reduces the effect of the direct response and low frequency drifts (see the Methods). The filtered data trace is then convolved with all the matched filters from the bank, with potential EPSCs having better fitting of the templates and exhibiting larger convolution amplitudes. The examples of matched filters and their convolution traces are shown in Figure 2A and B. Note that the filters have different shapes or waveforms, based upon a range of EPSC templates selected from experimental datasets. For each candidate EPSC, given that multiple samples of a convolution trace from one matched filter are likely to exceed the threshold, and considering that multiple convolution traces can exceed the threshold, the centers of mass of all the suprathreshold segments in all convolution traces are calculated. The arrival time of candidate EPSCs can be found in the vicinity of the center-of- mass points (see the Methods for details).

The EPSCs detected above need to be subjected to additional tests. To exclude direct responses, candidate EPSCs with their arrival times occurring within the direct response window (within 10 ms of the laser onset) are dismissed. While high-pass filtering reduces the direct response amplitude and duration, its convolution trace may still exhibit extremely large values (as much as 10 times greater than those of indirect synaptic responses) with long durations. With this consideration, within 30 ms of the laser onset, candidate EPSCs are declared eligible only if the convolution traces remain below the outlier threshold, but exceed the detection threshold. Detected events that fail this test are excluded from the list of candidates (Figure 2A). On the other hand, certain direct responses (e.g., those from the proximal or apical dendrites, see Figure 1) are relatively small, and their convolution traces may not exceed the outlier threshold. However, these direct responses can be correctly identified (Figure 2B), because their leading edge is traced back to the 10 ms direct response window.

The over-riding synaptic events are typically superimposed on the trailing part of the direct response (defined as the points between the (negative) peak of the direct response and the return to the baseline). While the aforementioned detection algorithm detects small, over-riding synaptic events, it also detects such responses that exhibit inflection points or “EPSC-like” notches that are related to baseline fluctuations. To eliminate these events from candidate EPSCs, an amplitude check is performed by comparing amplitudes of candidate EPSCs to a pre-set threshold based upon the mean amplitude of spontaneous EPSCs (assessed in the training stage). Normally, the cut-off threshold is based on statistical parameters estimated during the template training procedure. However, for detection of weak EPSCs, the cutoff threshold can be empirically set based upon the spontaneous EPSC level. Candidate events that do not get excluded by the above additional criteria represent detected EPSCs.

### Detection performance evaluation with simulated neural data

**Figure 3:**
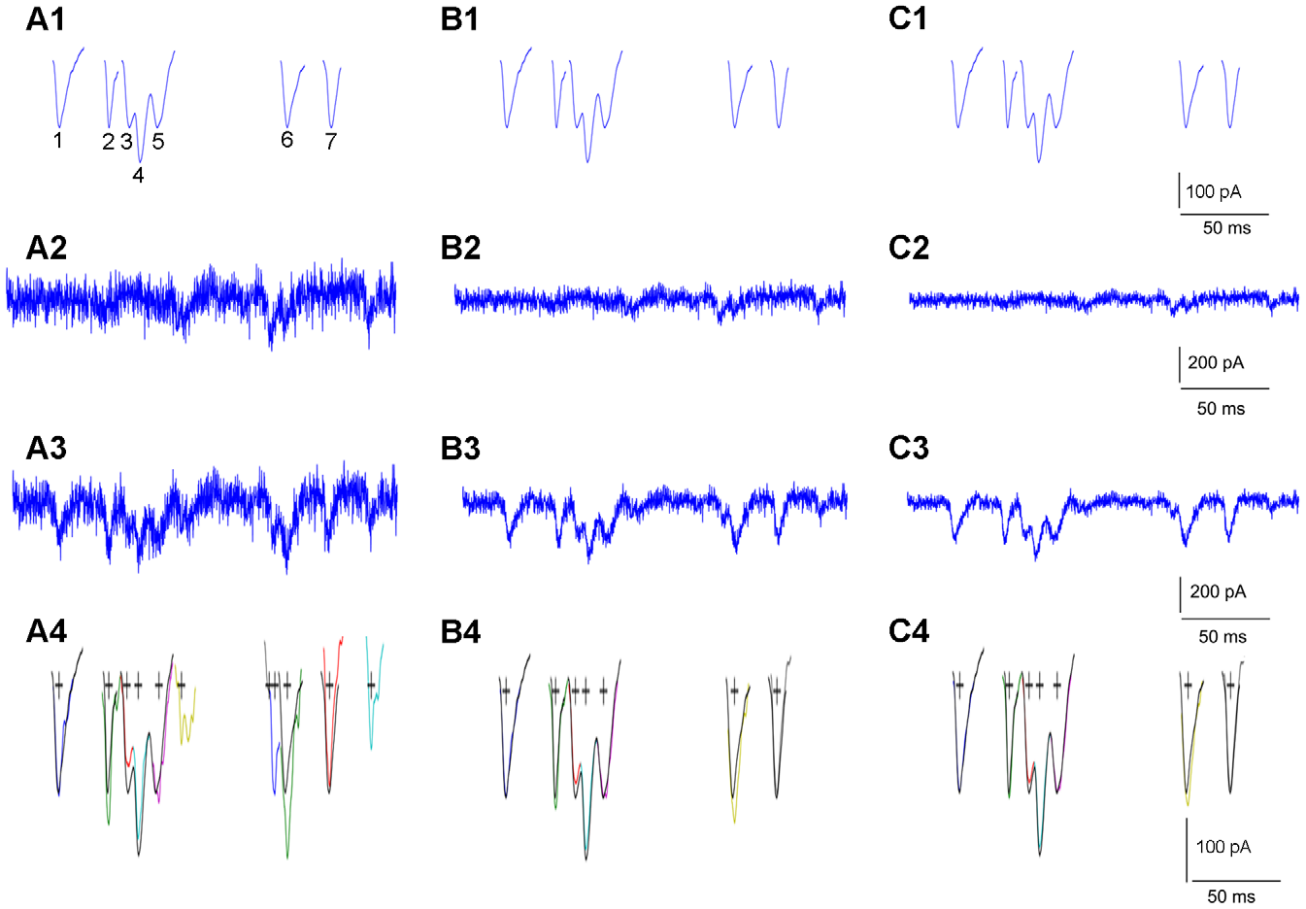
Simulated neural data and detection examples. A1, B1 and C1 are the same set of test templates (EPSC examples) acquired from experimental recordings, normalized to their peak amplitudes. Within this set of templates, simulated EPSCs of 1, 2, 6 and 7 are distributed as individual, non-overlapping events, while simulated EPSCs of 3, 4 and 5 overlap and take place as one complex and overlapping response. A2, B2 and C2 are the baseline spontaneous activity (noise) trace, with noise variances scaled to the test template amplitude with different SNRs. A3, B3 and C3 are simulated data traces by superimposing the template events with different degrees of noise. A4, B4 and C4 show EPSC detection results (color coded, with the estimated arrival time marked as ‘+’) through convolving the simulated data traces in A3, B3 and C3 with 10 matched filters, respectively. The original EPSC template events (black, as shown in A1, B1, C1), are plotted for evaluating software detection. The detection procedure uses a detection threshold at μ+3σ (3 standard deviations from the mean of the maximal template convolution values of the filters), and uses an amplitude cutoff of 60 pA, which is about 25% of the peak values of the individual test templates.

Since in actual recording experiments, the number of synaptic events and their exact arrival times (“ground truth”) are not perfectly known, the performance of our method was first assessed on simulated data (Figure 3). This allowed us to systematically vary the parameters critical for detection, such as SNR and detection thresholds, and evaluate the performance in terms of the probability of correct detection (Pcd) and probability of false alarm (Pfa).

**Figure 4:**
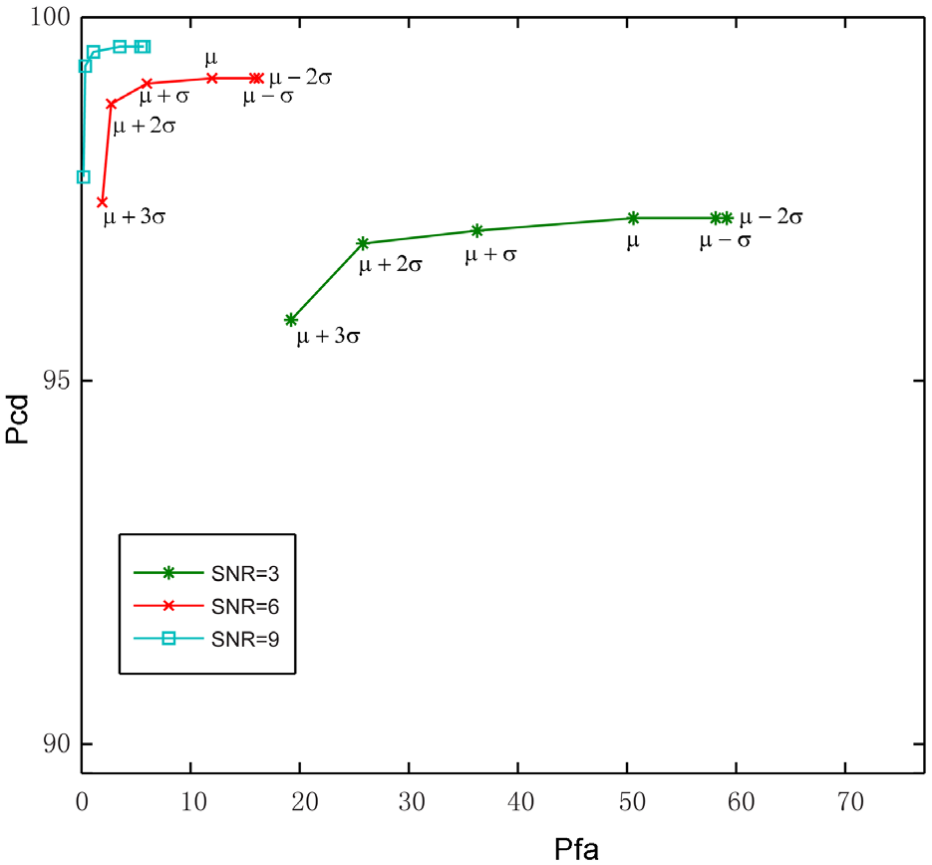
Detection performance evaluation on simulated data by using Receiver Operating Characteristic (ROC) curve analysis. The horizontal axis shows the probability of false alarm (Pfa), and the vertical axis shows the probability of correct detection (Pcd). Each ROC curve represents the software PSC detection performance at a fixed SNR (3, 6 or 9) with different detection thresholds. The detection thresholds ranges from −2σ to 3σ from the mean of the maximal template convolution values. The ROC curve for each combination of the detection threshold and SNR was calculated by averaging the performance over 200 trials.

Our technique was tested under different SNR and detection threshold scenarios. To ensure statistically meaningful results, for each SNR value, 200 independent Monte Carlo trials were performed, and the technique was applied by varying the detection threshold values between 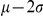 and 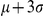 (in increments of 1*σ*), where *μ* and *σ* are the mean and standard deviation of the maximum convolution value *c*_max_ obtained in the training stage. Based on the detection results, Pfa and Pcd were calculated by averaging over trials, and plotted as receiver operating characteristic (ROC) curves in Figure 4. In all ROC curves, false alarms and correct detection are traded off at varying threshold values. Depending on the cost associated with omission and false alarm errors, the optimal detection threshold can be set. At low SNRs, the ROC curves are more spread for the detection thresholds chosen around the mean, *μ*, indicating higher sensitivity to the choice of threshold. Conversely, at SNR≥9, a situation likely to be found in actual recordings, the choice of threshold is less critical, as performances tend to cluster around the optimal point (Pfa = 0, Pcd = 1).

By analyzing the estimated arrival times of the correctly detected EPSCs, we found that on average the estimated and the true arrival time differed by 0.15±0.49 (mean ± SD), 0.02±0.32, and 0.0±0.24 ms, for SNR = 3, 6, and 9, respectively, which is insignificant compared to the typical duration of the template EPSCs. Based on these results, as well as the results from the ROC curves, especially at high SNR values, we conclude that our method is expected to perform well in experimental conditions.

### Analysis of experimental data, in comparison with human detection performance

**Figure 5:**
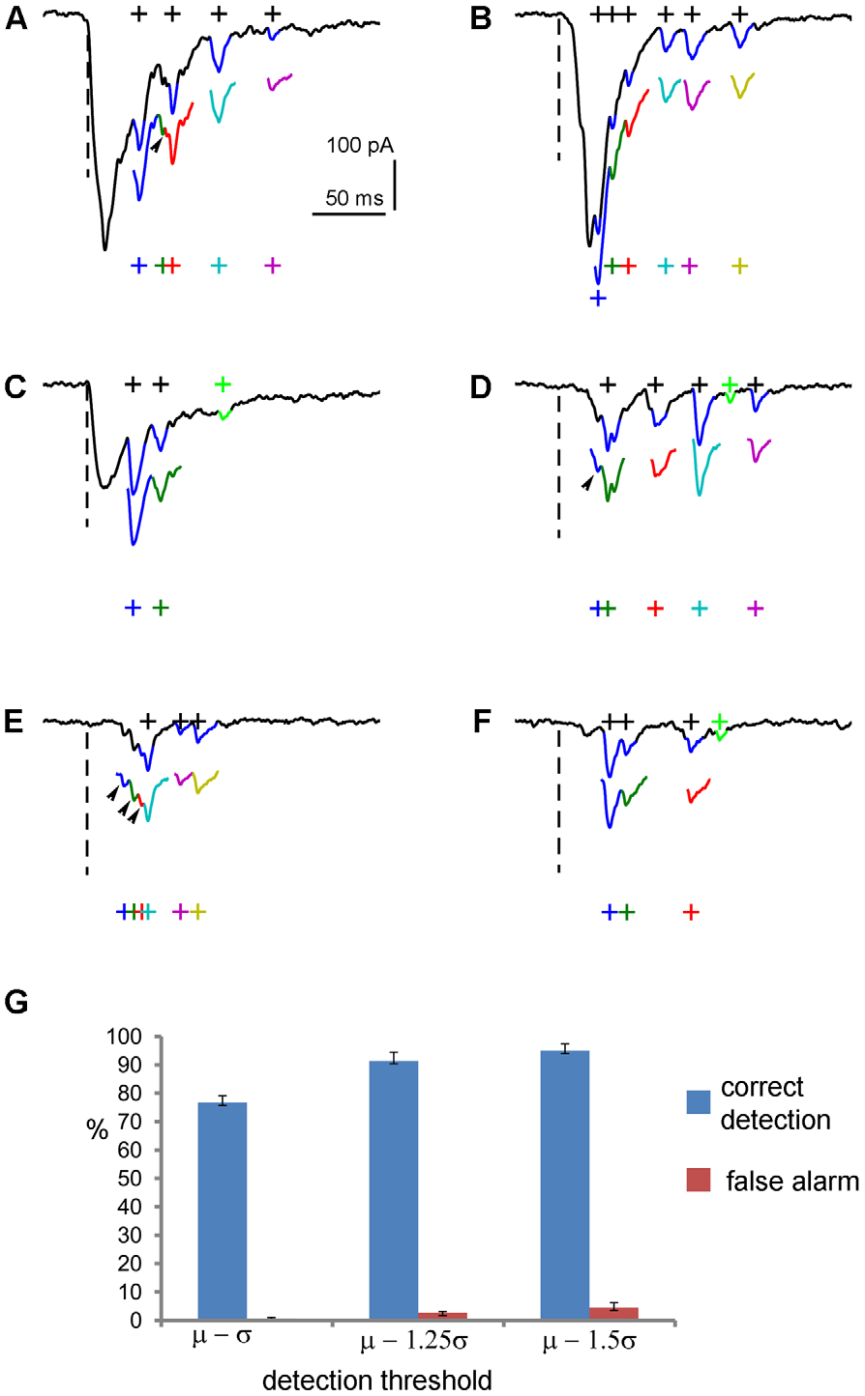
Analysis of experimental data through matched filtering. A–F: Typical examples of detection and extraction of photostimulation-evoked EPSCs, in comparison with human visual detection. The raw data traces were shown in solid back, with the overlaying segments of EPSCs (blue) identified by an experienced human operator. The black crosses indicate the center of the human selected EPSCs. The color-coded segments shown below the raw data traces are detected and individually extracted EPSCs through matched filtering, with the respective crosses indicating the detected EPSC centers. The arrows and arrow heads point to the extra events correctly detected by the software, but missed by the human. The weak EPSCs (with the amplitudes of <20 pA, about the spontaneous EPSC level) (green) identified by the human are missed by the automated detection, because of the pre-set amplitude cutoff (20 pA). G: the bar graph summarizing the percentage of correct detection and percentage of false alarm of the automated detection of EPSCs across different data sets (N = 3), using the same filter bank at multiple detection thresholds (μ−1σ, μ−1.25σ and μ−1.5σ). The values are presented as mean ± SE. Each data set contained more than 200 photostimulation-evoked EPSCs, and the detection results were inspected and verified by a human operator.

Our new technique was further validated on experimental data, while compared to that of manual (human) detection. Typical examples of software detection and extraction of photostimulation-evoked EPSCs, along with human visual detection of these events, are illustrated in Figure 5 A–F. These data traces include direct responses and synaptically mediated EPSCs, and contain complex overlapping events. In most occasions, EPSCs detected by the software and the human operator matched quite well, with software detection performing better than the human in identification of overlapping synaptic events (see the arrow heads in Figure 5). It should be noted that some of the weak EPSCs (with the amplitudes of about the spontaneous EPSC level) identified by the human, however, were missed by the automated detection, because of the pre-set cutoff threshold for evoked EPSC amplitudes. The inclusion of the amplitude cut-off setting is necessary for rejecting noise-related artifacts, due to an inherent trade-off between the sensitivity and specificity of our and any other statistical detection method [15]. Those missed weak EPSCs were proportionally insignificant, as they accounted for less than 4% of all the candidate events across individual datasets. In addition, considering that the software detects both the spontaneous baseline synaptic activity and photostimulation responses, and as the baseline spontaneous response is subtracted from the photostimulation response, the missed measurement of weak EPSCs at the spontaneous level does not have a major impact on our final measurement and analysis of EPSCs across many photostimulation sites (data not shown). Figure 5G summarizes quantitative evaluations of the automated detection of EPSCs, using the same filter bank at multiple detection thresholds. In general, the method performance was excellent and stable across different data sets. With the detection results inspected and verified by experienced human operators, the average probability of correct detection (Pcd) is 87.7%, with the average false alarm (Pfa) rate of 2.6% for the three detection thresholds chosen as 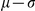, 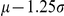 and 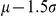. Specifically, the probability of correct detection is 76.7%±2.4% (mean ± SE), 91.45%±3.1%, and 94.9%±2.43% respectively; the corresponding probability of false alarm is 0.67%±0.37%, 2.72%±0.44%, and 4.43%±1.75%, respectively. In practical settings, our software implementation includes quick tests of selected data traces to determine appropriate detection thresholds.

In addition, the accuracy of this technique did not seem to depend much on the training stage and the choice of EPSCs for the design of the filter bank. To test the robustness of the method with the template design variability, a human operator repeated the filter design process by selecting a different set of EPSCs and consequently obtaining a different set of templates. When this template set was used for automated detection of EPSCs across the same data used for Figure 5G, the overall rates of correct detection and false alarm were 91.7% and 7.3%, respectively, similar to the rates reported with the first template set. Stable results were also obtained from a template set from a different operator, as the overall rates of correct detection and false alarm for the same dataset were 88.6% and 5.2%, respectively.

**Figure 6:**
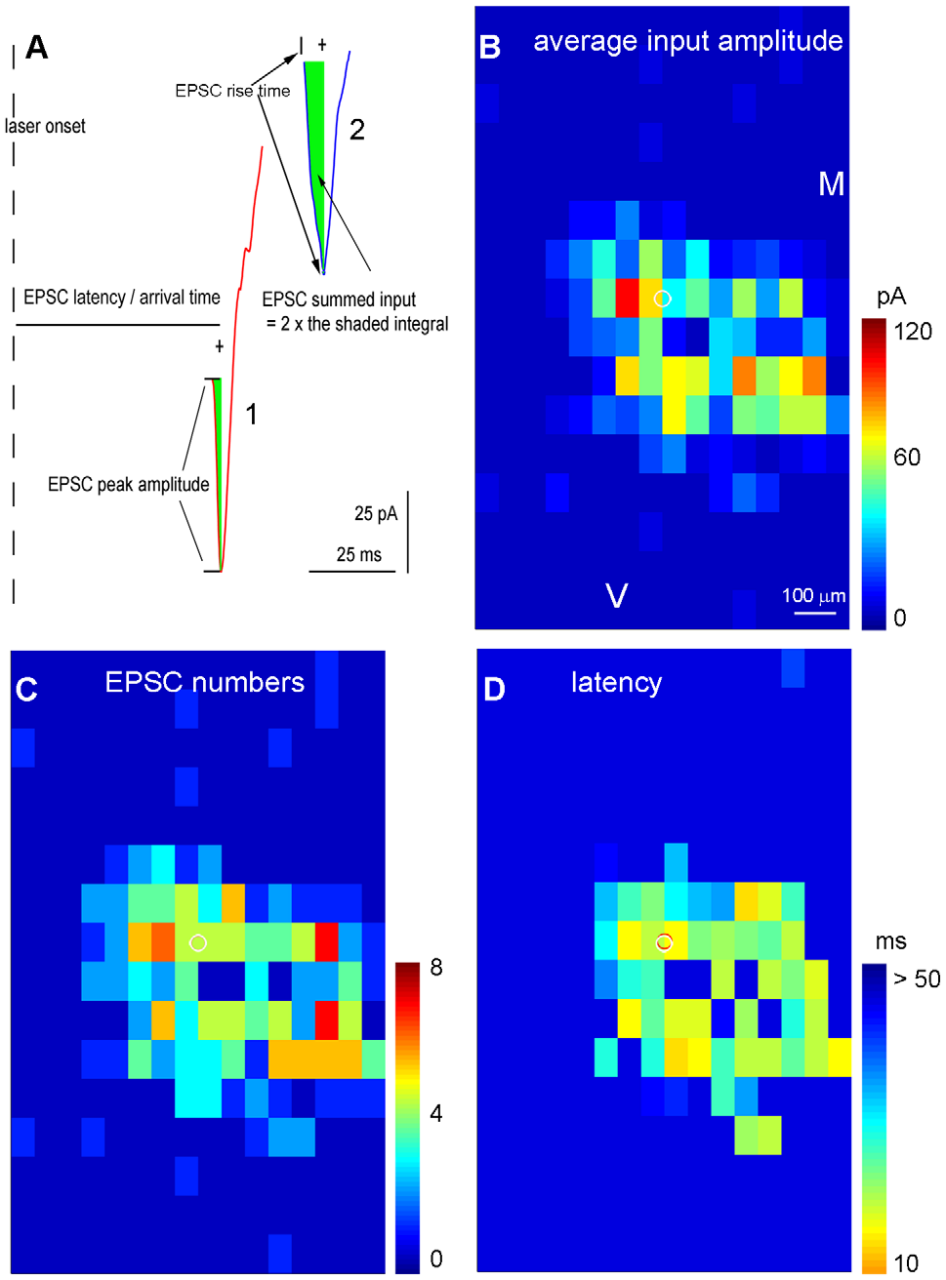
EPSC analysis and photostimulation data map construction. A shows the two extracted EPSCs (1, 2), one showing an example of over-riding EPSCs on the direct response, and the other showing an EPSC without being affected by the direct response. As illustrated in the two examples, individual EPSC peak amplitudes and summed input amplitudes, the EPSC rise time (from the onset to the peak time), EPSC latency/arrival time, and the number of EPSCs per site are measured. As the trailing portion of the over-riding EPSC can be skewed by the direct response, the individual EPSC summed input is defined as 2×[the integral area between the leading edge and the EPSC center] (the green shaded area). B, C, D are the color-coded maps (16×16 sites) of average input amplitude, the EPSC numbers, and the first detected EPSC latency per site, respectively, for the data set shown in Figure 1. The average input amplitude from each stimulation site is the mean amplitude of EPSCs in the response analysis window, with the baseline spontaneous response subtracted from the photostimulation response of the same site. The calculation is based upon the measurement of the total sum of individual EPSCs from each photostimulation site for the specified analysis window, and the value is expressed as picoamperes (pA). The number of EPSCs and the arrival time or latency of the first detected EPSC per site are also measured and plotted. M: medial; V: ventral.

After correct detection and extraction of the events, EPSCs are subsequently analyzed and the parameters such as EPSC peak amplitudes and summed input amplitudes, EPSC rise times, EPSC latency/arrival times, and the number of EPSCs from each photostimulation site are measured (Figure 6 A). As the trailing portion of the over-riding EPSC is often skewed by the direct response, the individual EPSC summed input is defined as 2×[the integral over the segment between the leading edge and the EPSC center]. For the purpose of visual display, a color-coded map is constructed to illustrate the pattern of excitatory input to the recorded neuron (Figure 6B). The number of EPSCs and the arrival time or latency of the first detected EPSC per site are also measured and plotted (Figure 6C and D).

Our automated procedure was much faster and more efficient than human detection. It is estimated that detection and analysis of photostimulation-evoked EPSCs with the software implementing our novel detection method are at least an order of magnitude faster than the human manual detection and analysis. Thus, this new technical advancement can greatly facilitate data analysis for photostimulation and other similar experiments.

### Characterization of photostimulation-evoked EPSCs

**Figure 7:**
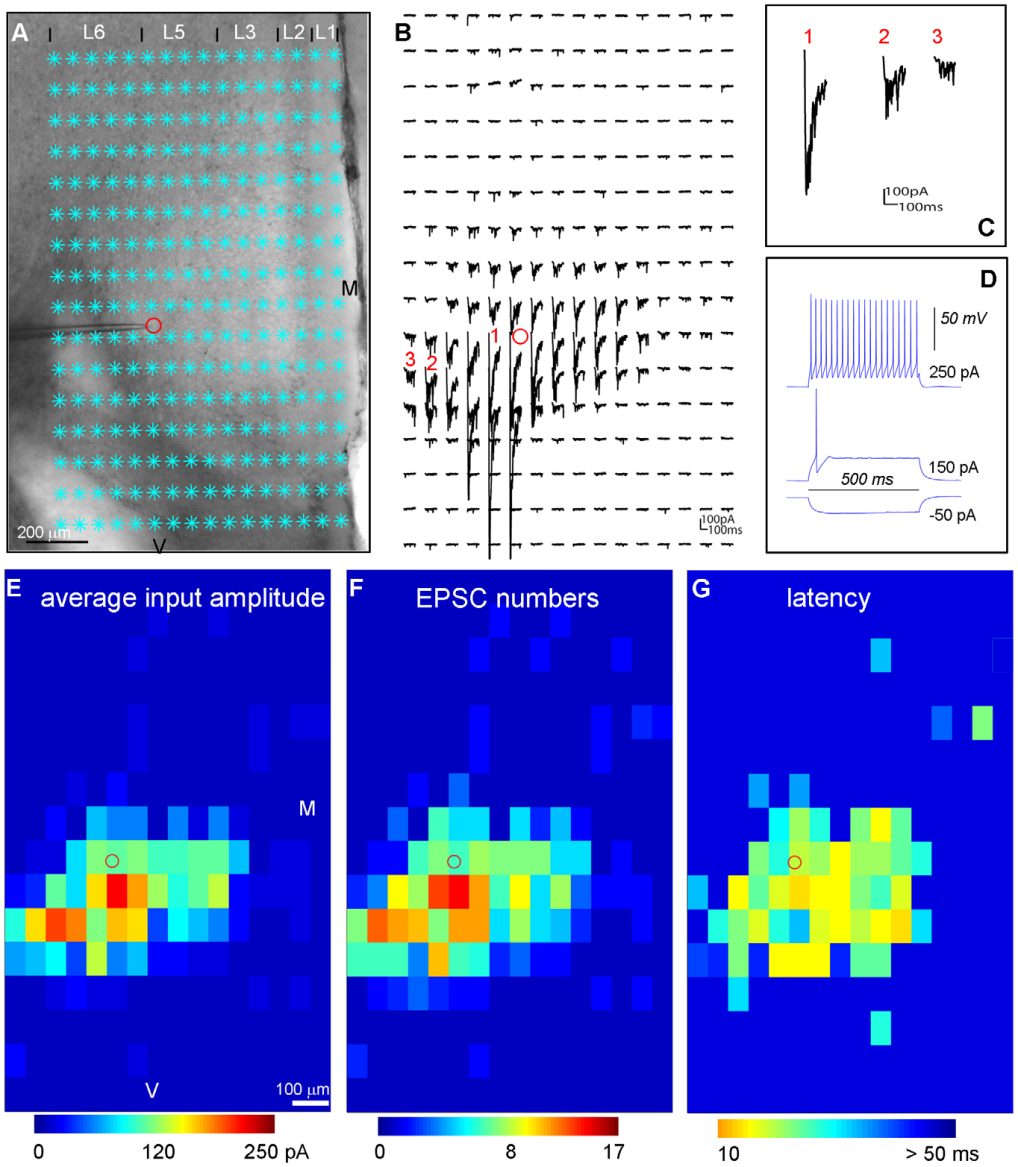
Excitatory input of local circuits to a fast-spiking (FS) inhibitory cell. A–D are similarly formatted as in Figure 1. A shows a mouse prefrontal cortical slice image with the superimposed photostimulation sites (cyan stars) across all the cortical layers 1, 2, 3, 5 and 6, with a glass electrode recording from a fast spiking inhibitory interneuron in the border of layers 5 and 6 of the prelimbic area in prefrontal cortex. The red circle indicates the cell body location. M denotes medial, and V denotes ventral. B shows an array of photostimulation-evoked response traces from the locations shown in A, with the cell held at −70 mV in voltage clamp mode to detect inward excitatory synaptic input. Examples of photostimulation-evoked responses are illustrated by the traces of 1, 2 and 3, which are expanded and separately shown in C. Trace 1 is an example of direct response with over-riding synaptic responses. Traces 2 and 3 are typical examples of synaptic input responses of FS cells. D shows the FS cell's intrinsic firing pattern with its voltage response traces to current injections at different amplitudes of −50, 150 and 250 pA, respectively. E, F and G present the color-coded maps of average input amplitude, the EPSC numbers, and the first detected EPSC latency per site, respectively, for the data set shown in B.

Given that EPSCs recorded from different cell types may differ in their strength and kinetics in mouse sensory cortex [2], in this study we further quantified and compared the EPSCs obtained from excitatory pyramidal cells and fast-spiking (FS) interneurons in mouse prefrontal cortex with our new technique. As illustrated in Figure 7, when compared to excitatory pyramidal cells (see Figure 1), FS cells tend to receive stronger and more frequent evoked EPSCs from local laminar circuits. In addition, FS cells' EPSCs may have faster kinetics, as they exhibit sharper rising phases. This qualitative impression was confirmed by our quantitative analysis of EPSCs recorded from these two cell types (Table 1). The data analysis was based upon automated detection and measurement of 689 photostimulation-evoked EPSCs recorded from excitatory pyramidal cells (N = 3), and 1076 evoked EPSCs recorded from FS cells (N = 3). As seen from Table 1, excitatory pyramidal cells had weaker EPSCs than FS cells, as established by comparing their median EPSC peak amplitudes which were 35.66±2.31 pA (mean ± SE) and 50.77±2.84 pA, respectively. Compared to excitatory pyramidal cells, the EPSCs of FS cells had on average shorter rise times, as their respective values were 2.93±0.73 ms (FS cells) and 4.7±0.31 ms. Excitatory and FS cells also differed in their average EPSC frequencies per stimulation site, as their respective values are 4.37±0.59 Hz and 7.87±1.49 Hz. Finally, the latencies of the first detected EPSC per site for excitatory pyramidal and FS cells were relatively similar (42.4±5.23 ms vs 37.9±4.82 ms). Therefore, our novel technique allows detailed quantitative data analysis and enables efficient treatment of large datasets through dependable, automated detection and characterization of synaptic events.

**Table 1:** Quantitative analysis of photostimulation-evoked EPSCs recorded from excitatory pyramidal cells and FS cells.

	EPSC peak amplitude (median, pA)	EPSC rise time (median, ms)	mean EPSC frequency (Hz) across photostimulation sites	The latency of first detected EPSC per site (median, ms)
**excitatory pyramidal cells**	35.66±2.31 (mean ± SE)	4.7±0.31	4.37±0.59	42.4±5.23
**fast-spiking (FS) inhibitory cells**	50.77±2.84	2.93±0.73	7.87±1.49	37.9±4.82

Note that the data summary is based upon automated detection and measurement of 689 and 1076 evoked EPSCs from excitatory pyramidal cells and FS cells (N = 3 each) recorded in the deep layers of the prelimbic area, respectively.

### Application of the method to IPSC detection

**Figure 8:**
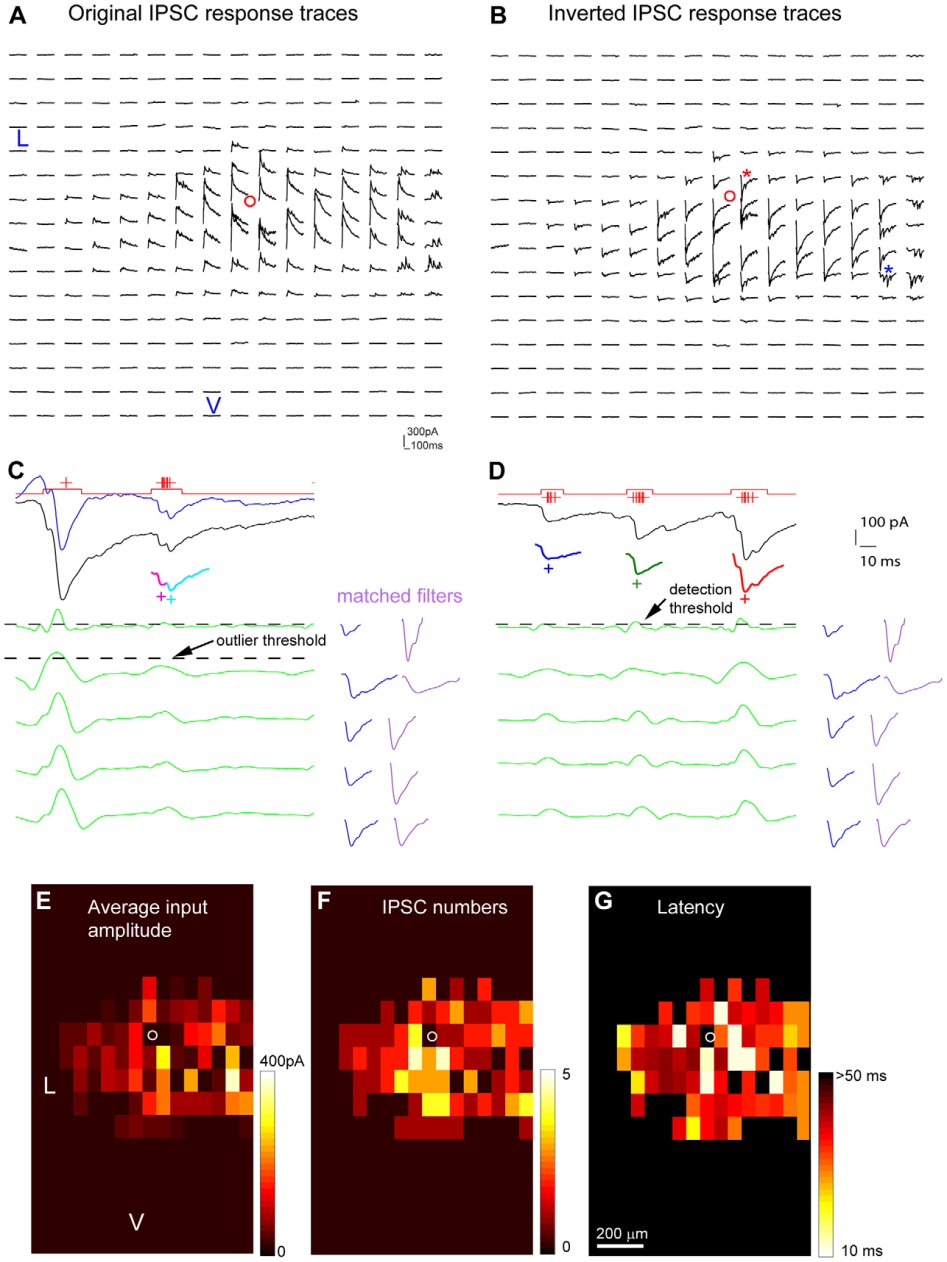
Extension of the method to the detection of IPSCs with matched filters. A and B are the original and sign-inverted IPSC response traces, respectively, which were from a layer 5 pyramidal neuron in the prelimbic area of mouse prefrontal cortex. The small red circles in A and B indicate the location of the recorded cell body. C and D are the illustration of matched-filtering detection of inverted IPSCs, reminiscent of EPSC detection (See Figure 2). The data traces for C and D are from the map sites indicated by the red and blue stars in B, respectively. The black traces are raw signals and the blue one shown in C is a high-pass filtered signal trace. In C and D, five exemplary convolution traces (green) produced with five matched filters (purple) are shown. The original EPSC templates (blue) used to synthesize the matched filters are also shown next to the convolution traces. The data trace in C has one large direct response, superimposed with two IPSCs that are color coded and individually extracted (shown below the original trace with the crosses indicating the event peaks), while the data trace in D contains three IPSC events (color coded and individually extracted, shown below the original trace). See Figure 2 for other conventions. E, F and G are the color-coded maps of average input amplitude, the IPSC numbers, and the first detected IPSC latency per site, respectively, for the raw data map shown in A. The small white circles indicate the location of the recorded cell body. L: lateral; V: ventral. The average input amplitude in each stimulation site is the mean amplitude of IPSCs in the response analysis window, with the baseline spontaneous response subtracted from the photostimulation response of the same site. The number of IPSCs and the arrival time or latency of the first detected IPSC per site are also measured and plotted.

Given the general applicability of our matched filter detection and extraction algorithms, our method can be appropriately modified and further applied to detection and extraction of other types of electrophysiological signals. For example, the technique has been easily modified to accommodate detection and analysis of inhibitory postsynaptic current (IPSC) responses. As illustrated in Figure 8A and B, for the IPSC detection, we first inverted the sign of IPSC responses, so the outward IPSC responses turned into EPSC-like inward responses. Note that compared to EPSCs, inverted IPSCs tend to have different waveforms with longer response durations (see Figures 1 and 8). As done in EPSC detection, the bank of matched filters was then generated based upon the inverted IPSCs and automated detection was applied for IPSC map data analysis and plotting (Figure 8C–G). Similar to EPSC detection, our method achieved excellent performance in IPSC detection across datasets.

## Discussion

In this study, we have developed a novel matched filtering technique for automated detection and extraction of synaptic events by combining the design of a bank of matched filters with the detection and estimation theory. The current technique has overcome the limitations of previously described threshold and template comparison techniques in detection of complex evoked synaptic signals with variable amplitudes and superimposed events.

An important novel feature of our technique is utilization of a bank of matched filters for the detection stage, which offers several advantages over previous techniques of template comparison. When human supervision is allowed, the optimal detector is a matched filter (template). Since humans have good understanding of the underlying signals, synaptic events can be reliably selected and their waveform appropriately modeled with high order polynomials (templates). EPSCs that match the templates are detected with high sensitivity by convolving with templates; artifacts and noise transients are rejected (filtered out) because they do not match the template waveform and time course. However, previous techniques using a template with fixed or variable amplitudes resulted in low sensitivity if the actual event waveform deviated from the template waveform; these techniques were not as effective for detecting overlapping events and compounds of events with different kinetics [10], [12]. Compared to fixed or scaled templates, even a few templates clearly increase the sensitivity of EPSC detection [14]. This major issue of single template comparison has been avoided in our new technique, as an array of filters based upon identified EPSCs from experimental data in the training stage provides a range of templates with variable shapes and durations that potentially match a variety of EPSCs found in experimental recordings.

For the design of filters, the training stage requires prior knowledge about evoked EPSCs and requires human supervision in selecting typical events for matched filter synthesis. However, the training stage is rather quick (∼10 min), and templates and parameters acquired from a typical data set can be used for detection of EPSCs in other similar experiments. In addition, the bank of multiple templates allow the statistics of the expected event waveform characteristics and time courses to be derived, and detection thresholds and safeguards against false detection to be subsequently set in a statistically meaningful manner. This constitutes one important novel of our method, as in previous studies the criteria used for both detection and extraction were mostly set empirically through error and trial [5], [9], [10], [11], [12], [14].

Although the present study was focused on EPSC detection and extraction, considering the general-purpose nature of our matched filtering and signal recognition algorithms, we expect the technique to be applicable to detection and extraction of other electrophysiological events such as extracellular action potentials, and event-related local field and electroencephalogram potentials as well as optical imaging signals (e.g., calcium indicator signals and voltage sensitive dye signals) in general. This generalizability follows from the theoretical properties of the matched filter which is known to be the SNR-optimal signal detector [15]. Clearly, the application of our technique to other domains will require modifications, including the design of an appropriate filter bank and adjustment of sensitivity/specificity thresholds. These modifications, however, are rather easy to implement using our user-friendly software. For example, our technique has been further applied to the detection and analysis of IPSC responses. To facilitate IPSC detection, as EPSCs and IPSCs have different signs, our method simply inverted the polarity of the original raw data traces, and the filter design and automated detection steps were applied in the same way as done in the EPSC detection. As for the detection of extracellular action potentials, the use of single or limited waveform templates has been used in prior studies [26], but the efficiency and sensitivity of detection can be greatly improved with the design of a bank of matched filters, as done in the present study. Moreover, similar to EPSCs or IPSCs, optical imaging signals such as calcium transient signals and fast voltage sensitive dye signals are mostly unipolar [16], [27] and have varying amplitudes and overlapping events. Therefore, as illustrated in the IPSC detection, the adoption of our new method to detection of optical signal events should be relatively simple. Finally, we hypothesize that our technique can be modified to accommodate detection of event-related local field and electroencephalogram potentials. Similar to extracellular action potentials, these usually have biphasic (bipolar) waveforms, and while modification procedures are likely to be different from those used in IPSC detection, the general algorithms can still be applied.

Another innovation of our technique is that convolution traces of the matched filters are compared to an event detection threshold to construct suprathreshold segments of the data trace, and the center of mass of each of the convolution trace is found and declared as an occurrence time candidate of an EPSC. Single or multiple overlapping EPSCs within each suprathreshold segment can be correctly identified (see Figure 5). Our algorithm manages to detect multiple or pairs of events that are separated in time by less than the length of the templates, which previous template comparison techniques would not be able to (e.g., see Clements and Bekk, 1997). Moreover, with additional constraints of the direct response and outlier windows, our technique is able to exclude direct photostimulation responses and detect synaptically mediated EPSCs over-riding on the direct response. Detection and extraction of this type of over-riding events illustrates the power and effectiveness of our new technique, as previously published techniques would fail in such complex situations [9], [10], [12], [13], [14].

Our results show that the new method can identify events with high sensitivity and a low false alarm rate, with tests on both simulated data and experimental data. In most occasions, the automated detection was at least as good as human visual event detection when applied to photostimulation experimental data. Our algorithm, in essence, only requires the user to select a set of typical synaptic responses from experimental data during the filter design/training stage in order to detect events, and obtain accurate estimates of the amplitude, timing and kinetic information of the detected events during the automated detection stage. In addition, if the default template library and threshold settings are used, the method can be implemented in a fully automated fashion. Should the default parameters prove inadequate, the efficient software implementation and fast execution of our method allow the parameter adjustment under training-derived statistical guidance.

With the established filter bank, the sensitivity and specificity of our technique is dependent on two parameters, the event detection threshold, and the event amplitude cut-off threshold. The statistics of the filter bank (e.g., the mean and standard deviations of convolution peak values) may help guide the setting of appropriate detection thresholds. In addition, the software implementation allows practical tests of selected data traces to determine optimal detection thresholds. As shown in our ROC analysis, the lower detection thresholds may present higher sensitivity in detection but with a higher false alarm rate. Sometimes when it is necessary to set a lower detection threshold for detecting low-amplitude events, the event amplitude cut-off threshold is important to reject noise-related artifacts, and ensures a low rate of false alarm.

In summary, our algorithms and software implementation enable dependable automatic detection of synaptic events with minimal human supervision. The use of a bank of matched filters and template-derived statistical guidance are important novel features of our technique. This work represents a substantial contribution to the recognition and detection of complex signals encountered in the studies of synaptic physiology.

## Supporting Information

Figure S1 **An explanatory flow-chart describing the general sequence of the application of matched filtering program to synaptic event detection.**
(TIF)

Figure S2 **The direct response and outlier windows, and detection and outlier thresholds.** Raw signal (black) showing direct response to the laser photostimulation (applied at 100 ms) and its return to baseline. Responses within the 10 ms window (*W_d_*, 100–110 ms) from laser onset were considered direct, as synaptic currents with such short latencies are not possible because they would have to occur before the generation of action potentials in photostimulated neurons. No EPSC events can be detected within the direct response window. High-pass filtered version of the response (blue) features a less prominent direct response. Note that phase shifts and shape distortions between the original and filtered traces are minimal, and so the occurrence times of EPSCs are preserved. Convolution traces (green) are obtained by convolving the high-pass filtered signal with 18 matched filters from the filter bank. Given that convolution traces may be affected by the direct response even after the direct response window, thereby yielding extremely large values (as the red arrow indicates), the convolution traces within this outlier window (*W_o_*, 110–130 ms) are compared to an addition (outlier) threshold (cyan line) and those that exceed this threshold in *W_o_* are dismissed as potential EPSCs. The green dashed line marks the detection threshold and red square pulses mark the suprathreshold segments (the segment of convolution trace above the threshold) for each convolution trace. The center of mass of each convolution trace within the suprathreshold segment is marked by a red dot, and declared as an EPSC occurrence time candidate.
(TIF)

Figure S3
**Identifying an EPSC peak through scoring criteria.** If multiple potential peaks in the original raw data trace are found in the vicinity of each occurrence time candidate, t_cm_, defined as 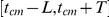 under each suprathreshold segment, they are scored according to three heuristic criteria, and the peak with the highest score is selected. For example, here are the two peaks (∼137 ms and ∼142 ms) found on the segment 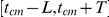, marked by dashed lines. The peaks are scored according to: the number of convolution traces that exceed the threshold at the peak time, the amplitude of the peak, and the value of the second derivative of the signal at the peak. All of these criteria are normalized between 0 and 100. The first criterion favors EPSCs whose shapes match multiple filters from the bank. More specifically, if the detected signal candidate has a “typical” EPSC shape captured by the bank of templates, it will have a high score by this criterion. The second criterion favors detection of EPSCs with higher amplitudes which typically provide higher SNRs. Finally, the second derivative criterion favors EPSCs which are peaky, and penalizes those which are irregular (flat), such as the peak at ∼142 ms. These criteria are weighted equally, and the occurrence time of the peak with the highest average score is selected. In this particular case, the scores of the first peak by the first, second and third criterion are 12, 71.46 and 0.45, respectively, while these respective scores for the second peak are 4, 65.84 and 0.16. The original value of the first score criterion is normalized with the total number of convolution traces, which is 18 in this case while the scores of other two criteria are normalized by each maximum value of all potential peaks. The normalized scores (at a scale of 0–100) of these two peaks by the first, second and third criterion are 66.67, 100, 100 and 22.22, 92.13, 35.71, respectively. The averaged overall scores are 88.89 and 50.02, respectively for the two peaks at ∼137 ms and ∼142 ms. Thus the peak at ∼137 ms is identified as an EPSC candidate (black cross).(TIF)

Figure S4
**Identification of the onset and offset time of the EPSC, detection of other potential EPSCs and noise rejection within** 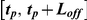 **.** After *t_p_* is identified, the onset and offset of estimated EPSC are to be found. The onset time is identified as the local maximum (first derivative crosses zero) or as the local supremum if there is no first derivative zero crossing on 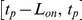. As shown in A, the offset time could be simply the local supremum if no local maxima exist within 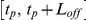. However, as shown in B, C, and D, if there is one or more local minima (t_p_ potential) on 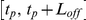, an additional measure is taken to locate the EPSC offset point. Considering that there may be potential EPSCs within 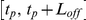, each local maximum (p_b_) on this segment could also represent the onset of the next potential EPSC. A simple amplitude test, where amplitude is defined as the difference of values at p_b_ and t_p_ potential in the original trace, is then performed; if the amplitude is greater than a pre-set amplitude threshold, p_b_ is used as the offset of the EPSC centered at t_p_ and the onset for the next potential EPSC centered at t_p_ potential (B). If the amplitude is less than the pre-set threshold, p_b_ is not considered the onset of the next potential EPSC, and the software continues its search for another local minimum. If there are no more local minima, the EPSC offset point is set at p_b_ or 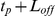, whichever point has a higher amplitude (C). If there are multiple local minima occurring after the potential EPSC boundary (p_b1_), the software compares the amplitude at p_b1_ and p_b2_ (D). If amplitude at p_b1_ is less than that at p_b2_, we use p_b2_ as the offset of the EPSC centered at t_p_ and as the onset of the next potential EPSC; otherwise p_b1_ is taken as the offset of the EPSC centered at t_p_, p_b2_ is ignored, and the search continues towards 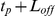.(TIF)
