# Supplementary figures and images for: Novel Use of Matched Filtering for Synaptic Event Detection and Extraction
Source: PLoS One. 2010 Nov 24;5(11):e15517. doi: 10.1371/journal.pone.0015517 (PMC2991367; doi:10.1371/journal.pone.0015517)

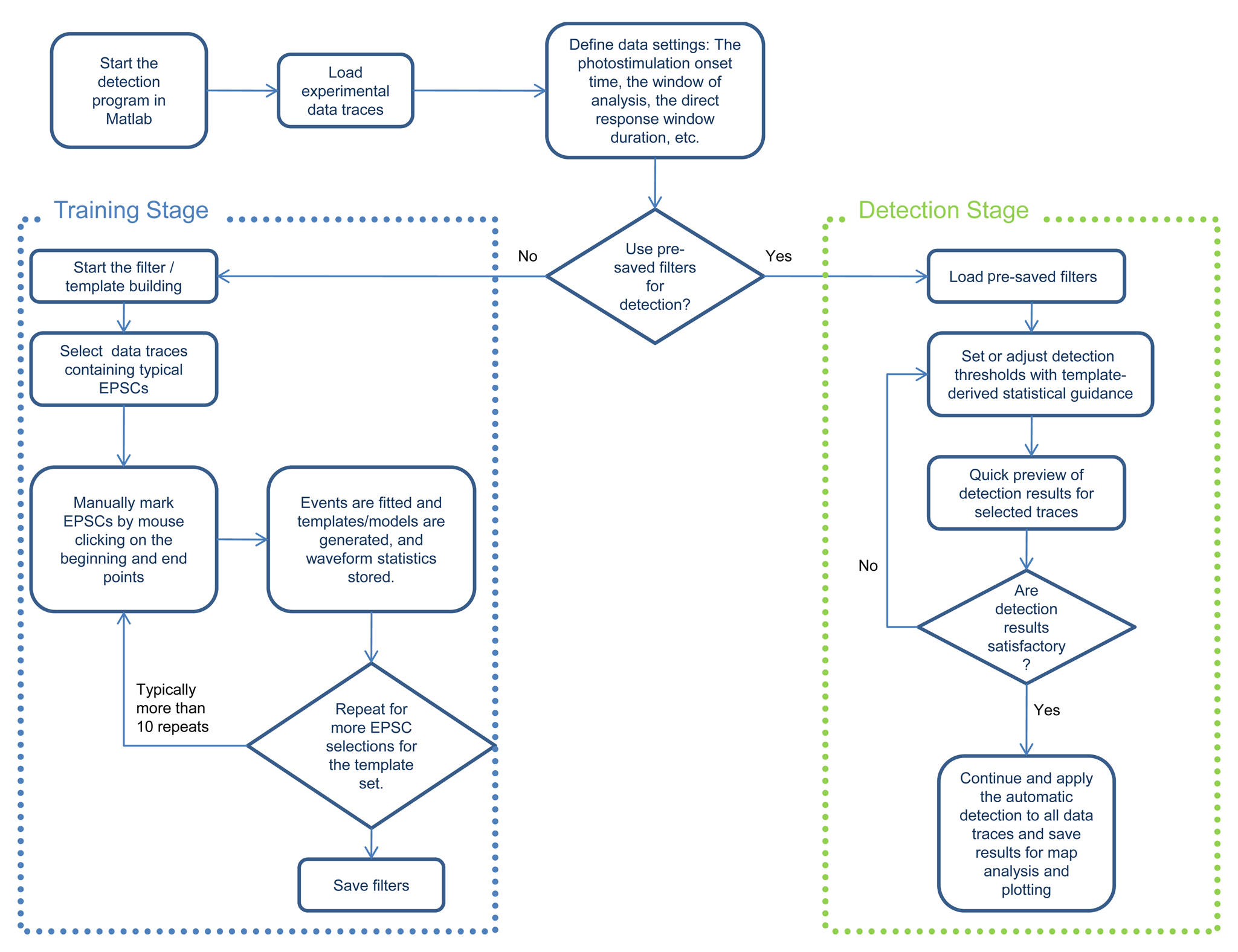

Supplement: Figure S1 — An explanatory flow-chart describing the general sequence of the application of matched filtering program to synaptic event detection. (TIF) [file pone.0015517.s001.tif]

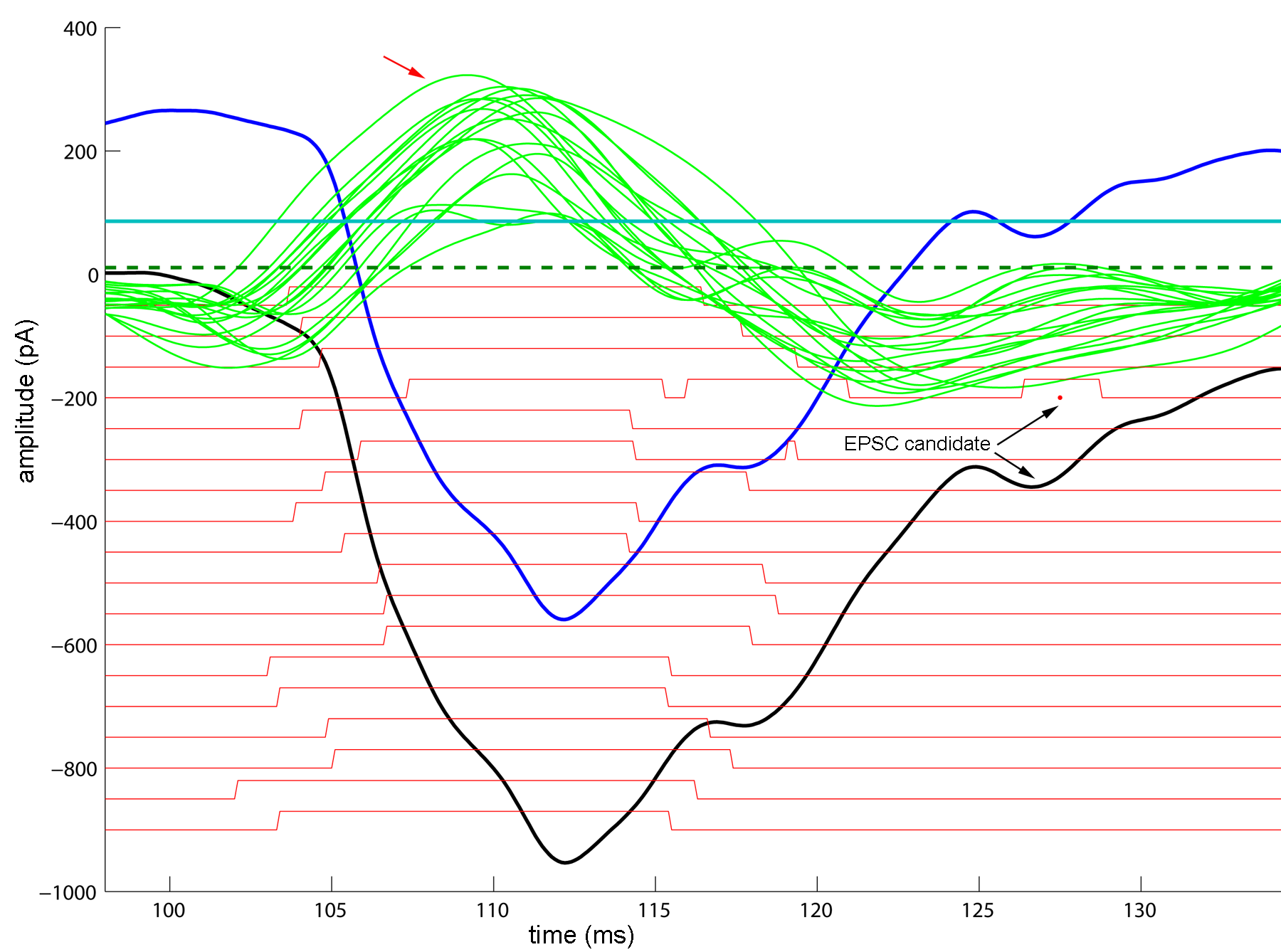

Supplement: Figure S2 — The direct response and outlier windows, and detection and outlier thresholds. Raw signal (black) showing direct response to the laser photostimulation (applied at 100 ms) and its return to baseline. Responses within the 10 ms window (Wd, 100–110 ms) from laser onset were considered direct, as synaptic currents with such short latencies are not possible because they would have to occur before the generation of action potentials in photostimulated neurons. No EPSC events can be detected within the direct response window. High-pass filtered version of the response (blue) features a less prominent direct response. Note that phase shifts and shape distortions between the original and filtered traces are minimal, and so the occurrence times of EPSCs are preserved. Convolution traces (green) are obtained by convolving the high-pass filtered signal with 18 matched filters from the filter bank. Given that convolution traces may be affected by the direct response even after the direct response window, thereby yielding extremely large values (as the red arrow indicates), the convolution traces within this outlier window (Wo, 110–130 ms) are compared to an addition (outlier) threshold (cyan line) and those that exceed this threshold in Wo are dismissed as potential EPSCs. The green dashed line marks the detection threshold and red square pulses mark the suprathreshold segments (the segment of convolution trace above the threshold) for each convolution trace. The center of mass of each convolution trace within the suprathreshold segment is marked by a red dot, and declared as an EPSC occurrence time candidate. (TIF) [file pone.0015517.s002.pot.tif]

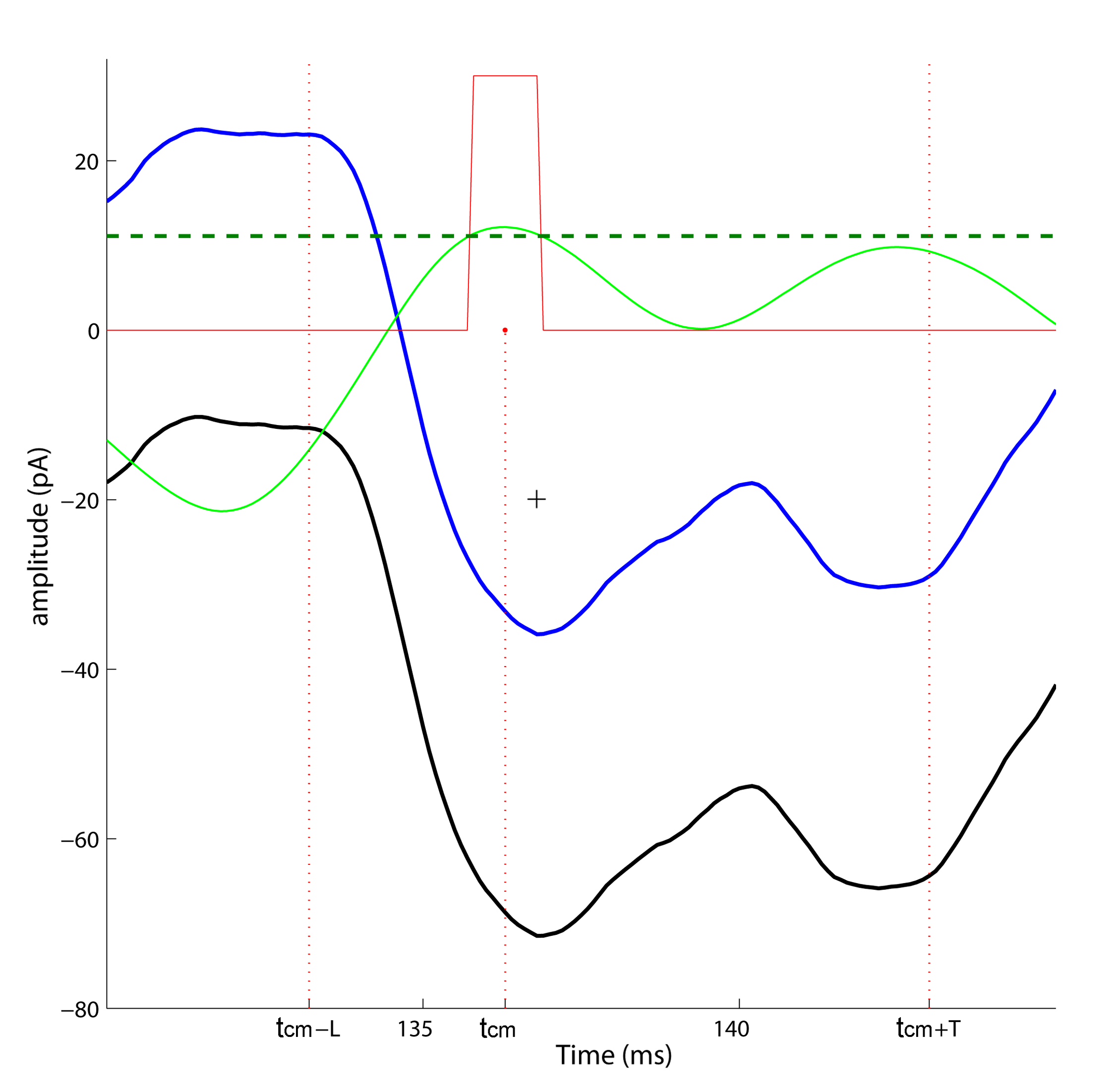

Supplement: Figure S3 — Identifying an EPSC peak through scoring criteria. If multiple potential peaks in the original raw data trace are found in the vicinity of each occurrence time candidate, tcm, defined as under each suprathreshold segment, they are scored according to three heuristic criteria, and the peak with the highest score is selected. For example, here are the two peaks (∼137 ms and ∼142 ms) found on the segment , marked by dashed lines. The peaks are scored according to: the number of convolution traces that exceed the threshold at the peak time, the amplitude of the peak, and the value of the second derivative of the signal at the peak. All of these criteria are normalized between 0 and 100. The first criterion favors EPSCs whose shapes match multiple filters from the bank. More specifically, if the detected signal candidate has a “typical” EPSC shape captured by the bank of templates, it will have a high score by this criterion. The second criterion favors detection of EPSCs with higher amplitudes which typically provide higher SNRs. Finally, the second derivative criterion favors EPSCs which are peaky, and penalizes those which are irregular (flat), such as the peak at ∼142 ms. These criteria are weighted equally, and the occurrence time of the peak with the highest average score is selected. In this particular case, the scores of the first peak by the first, second and third criterion are 12, 71.46 and 0.45, respectively, while these respective scores for the second peak are 4, 65.84 and 0.16. The original value of the first score criterion is normalized with the total number of convolution traces, which is 18 in this case while the scores of other two criteria are normalized by each maximum value of all potential peaks. The normalized scores (at a scale of 0–100) of these two peaks by the first, second and third criterion are 66.67, 100, 100 and 22.22, 92.13, 35.71, respectively. The averaged overall scores are 88.89 and 50.02, respectively for the two peaks at ∼137 ms a [file pone.0015517.s003.tif]

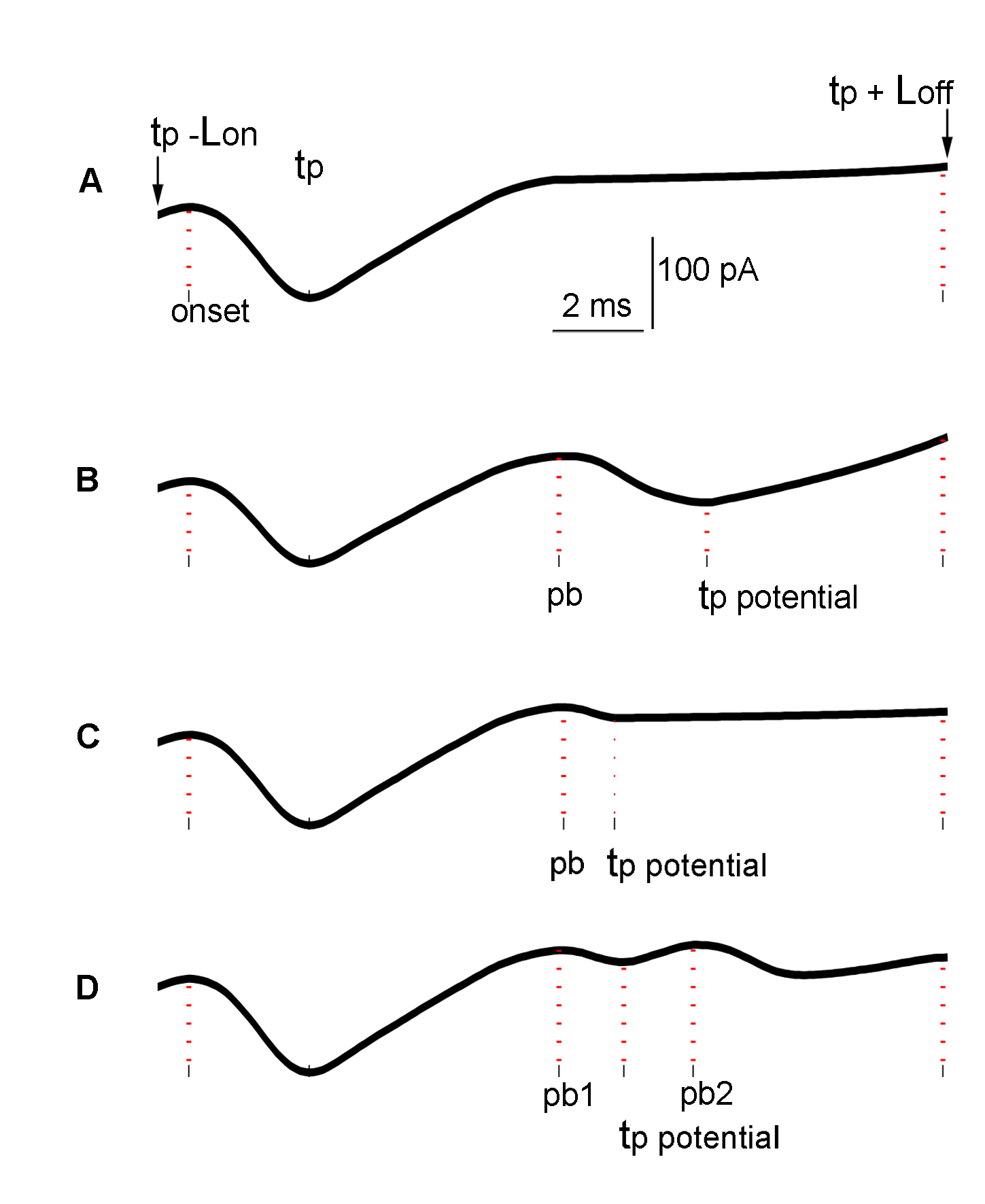

Supplement: Figure S4 — Identification of the onset and offset time of the EPSC, detection of other potential EPSCs and noise rejection within . After tp is identified, the onset and offset of estimated EPSC are to be found. The onset time is identified as the local maximum (first derivative crosses zero) or as the local supremum if there is no first derivative zero crossing on . As shown in A, the offset time could be simply the local supremum if no local maxima exist within . However, as shown in B, C, and D, if there is one or more local minima (tp potential) on , an additional measure is taken to locate the EPSC offset point. Considering that there may be potential EPSCs within , each local maximum (pb) on this segment could also represent the onset of the next potential EPSC. A simple amplitude test, where amplitude is defined as the difference of values at pb and tp potential in the original trace, is then performed; if the amplitude is greater than a pre-set amplitude threshold, pb is used as the offset of the EPSC centered at tp and the onset for the next potential EPSC centered at tp potential (B). If the amplitude is less than the pre-set threshold, pb is not considered the onset of the next potential EPSC, and the software continues its search for another local minimum. If there are no more local minima, the EPSC offset point is set at pb or , whichever point has a higher amplitude (C). If there are multiple local minima occurring after the potential EPSC boundary (pb1), the software compares the amplitude at pb1 and pb2 (D). If amplitude at pb1 is less than that at pb2, we use pb2 as the offset of the EPSC centered at tp and as the onset of the next potential EPSC; otherwise pb1 is taken as the offset of the EPSC centered at tp, pb2 is ignored, and the search continues towards . (TIF) [file pone.0015517.s004.tif]
